# Probiotics: Should All Patients Take Them?

**DOI:** 10.3390/microorganisms9122620

**Published:** 2021-12-18

**Authors:** Marta Katkowska, Katarzyna Garbacz, Aida Kusiak

**Affiliations:** 1 Department of Oral Microbiology, Medical Faculty, Medical University of Gdańsk, 80-204 Gdańsk, Poland; marta.katkowska@gumed.edu.pl; 2 Department of Periodontology and Oral Mucosa Diseases, Medical Faculty, Medical University of Gdańsk, 80-204 Gdańsk, Poland; akusiak@gumed.edu.pl

**Keywords:** probiotic, *Lactobacillus*, *Bifidobacterium*, infection

## Abstract

The usefulness of probiotics in the treatment as well as prevention of many infections and disorders has been confirmed by previous clinical studies. They can protect not only against gastrointestinal diseases such as diarrhea or enteritis but they have proven efficacy against pneumonia, urogenital infection, depression/anxiety, cancer metastasis, obesity, and others. However, it should be mentioned that not all clinical trials have shown improvement of health in patients undergoing probiotic treatment, and very rarely have even reported that probiotic strains may be the causative agents of opportunistic infections. Studies have documented cases of sepsis/bacteremia, endocarditis, liver abscess, pneumonia, and fungemia caused by probiotic strains, mainly in high-risk groups. This review summarizes the cases of infections caused by probiotic strains and the potential hazard associated with the supplementation of probiotics in seriously ill and hospitalized patients.

## 1. Introduction

Lactic acid bacteria (LAB) have long been known to have a positive impact on human health. More than a century ago, Metchnikoff hypothesized that consumption of LAB present in yoghurt can improve health and increase the lifespan of humans [1]. In 1965, Lilly and Stillwell used the word “probiotic” for the first time for substances that are secreted by one organism and exhibit a growth-promoting effect on another [2]. It is derived from Greek words *pro bios* meaning “promotion life” and is currently defined by the International Scientific Association for Probiotics and Prebiotics (ISAPP) as “live microorganisms that, when administered in adequate amounts, confer health benefits on the host” [3].

The most commonly used probiotic bacteria are LAB, such as *Lactobacillus* (now *Lacticaseibacillus*) and *Bifidobacterium*, which are indigenous members of the gastrointestinal microbiota in humans and popularly known as health promoters. *Bifidobacterium* was the first probiotic isolated by Henry Tissier in 1905 [4]. In 1983, Gorbach and Goldin first isolated *Lactobacillus* *rhamnosus* from the intestine of a healthy person (patented as *L. rhamnosus* GG) [5,6]. In addition to bacteria, yeasts, including *Saccharomyces cerevisiae* var. *boulardii*, which was discovered by Henri Boulard in 1920, are used as probiotics [2]. Probiotics are found in pharmaceutical preparations, dietary supplements, or fermented products. The Food and Agriculture Organization of the United Nations/World Health Organization (FAO/WHO) has suggested that the microorganisms that have been used safely for years in food and classified as “Generally Recognized as Safe” can be used as probiotics [7].

The beneficial effects of probiotics on health are well known [8]. The immunomodulatory activity of probiotics as well as the ability to modulate the intestinal barrier have a significant role in the prevention and therapy of infectious diseases and the inflammation of the digestive tract. Probiotic strains have a direct effect on commensal and pathogenic microorganisms colonizing the gut, which is important in the treatment and prevention of intestinal infections. They may also have the effect of restoring the microbiological balance in the host’s organism [4]. Probiotics can act on the binding of toxins produced by pathogenic microorganisms and inactivate them, which allows for detoxification of host and food components in the gut [5].

Consumption of probiotics concerns not only protection against gastrointestinal diseases but also includes the alleviation of disorders related to cardiovascular health [9], depression/anxiety [10], cancer metastasis [11], lowering cholesterol levels [12], type 2 diabetes [13], and obesity [14]. Protection against urogenital and respiratory tract diseases even nosocomial infections have also been attributed to probiotics [15,16,17,18,19]. Numerous studies have reported a positive effect of probiotics on the immune system, alleviating inflammatory or atopic disorders and allergies in infants [7,20,21,22].

Although the benefits of probiotic use are indisputable and widely proven, one cannot forget about exceptionally rare, but sometimes undesirable effects. Due to their widespread use, it is critical to make certain that the probiotic strains currently available on the market are safe to use [23]. Till now, no widely accepted guidelines related to probiotics use have been available, nor any regulation regarding their maximum dose that can be safely used [24,25]. Didari et al. concluded that probiotics can be safely taken by healthy individuals, whereas in seriously ill, hospitalized, postoperative, and immunocompromised patients, probiotics can potentially cause side effects [26]. Therefore, it is necessary to consider the risk–benefit ratio before prescribing probiotics for these populations [27]. Furthermore, it has been shown that, although very rarely, probiotic strains cause infections in humans [26,28,29].

This review summarizes the cases of infections caused by probiotic strains and the potential hazard associated with the supplementation of probiotics in seriously ill and hospitalized patients.

## 2. Safety and Standards Demonstrated by Probiotic Strains

The safety of probiotics is largely determined by the population who consumes them. In general, they are very well tolerated by healthy individuals, and the associated side effects are mild and do not pose a threat to health or life. On the other hand, in the higher risk group, there may be episodically severe side effects [29,30]. The FAO/WHO guidelines distinguish the adverse effects associated with the use of probiotics into four categories: systemic infections, adverse metabolic effects, excessive immune stimulation in susceptible persons, and transmission of antibiotic-resistance genes. Despite their widespread and long-term use and proven safety profile, probiotic strains should be constantly monitored for safety, as they can undergo possible genetic modifications [31].

There is no universally accepted system for regulating the use of probiotics, and only recommendations for their gradual evaluation have been proposed, including correct identification of the strain, in vitro research for the evaluation of properties, and in vivo studies on animals and humans for determining the safety of use and efficacy [32]. The European Food Safety Authority (EFSA) is in charge of regulating, assessing, as well as monitoring the safety of food and food supplements, including probiotic products, in the European Union. In Europe, the Qualified Presumption of Safety (QPS) list is currently used for evaluating the safety of probiotics [33]. In order to include a microorganism on the QPS list, the following criteria should be met: taxonomic determination of the strain, adequate information to determine safety, lack of pathogenic properties, and a well-defined purpose of use. The QPS status of microorganisms is assessed by the EFSA Panel on Biological Hazards. Since 2014, an assessment of microorganisms that are recommended for inclusion has been performed twice a year, and an assessment of microorganisms that are already included in the QPS list has been performed every 3 years. The absence of a microorganism on the QPS list does not imply that it is dangerous to health, nor does it exclude the possibility of using its products in the European Union (the long history of use and no adverse effects is sufficient), but it simply indicates that the microorganism has not been assessed by the EFSA [23].

Probiotics are used as dietary supplements (USA and Europe), food with specific health benefits (Japan), and natural health substances (Canada). In most countries, dietary supplements are required to meet much less stringent regulatory criteria, and are not subject to strict control or monitoring, unlike drugs, which are strictly regulated and monitored for safety, both before and after release on the market [23].

Reports of probiotic-related side effects have prompted some countries to tighten their regulations. For example, in 2009, Norway issued a warning against probiotic use by patients who are seriously ill, including those suffering from Antibiotic-Associated Diarrhae (AAD) or *Clostridioides difficile* infection [8]. In 2013, a group of Belgian experts issued the report “8651 Probiotics” after a detailed analysis of the issues associated with the use of probiotics. The report recommended that precise identification of the probiotic strain should be carried out, the safety of the strains in food should be assessed, and products containing probiotics should be labeled [34].

In addition to the international and national regulations, expert organizations provide opinions and guidelines on the use of probiotics, which constitute a valuable supplement to the practical aspects of using these strains. For instance, in 2006, experts from the Product Safety Forum of Europe (PROSAFE) provided recommendations for the safe use of probiotics. According to the guidelines, the probiotic strains that do not occur naturally (wild-type) are not allowed to be used as food additives for humans and animals, and strains containing genes of known and/or confirmed virulence are also prohibited. To evaluate whether probiotic strains are safe to use and verify the presence of possible pathogenic characteristics, in vivo analyses using animal models as well as randomized human colonization studies using a double-blind placebo are recommended as a reliable tool [35]. Similarly, in 2017, the Members of the European Society for Pediatric Gastroenterology, Hepatology and Nutrition (ESPGHAN) Working Group expressed their opinion on the need to develop and implement control methods for probiotic-containing products on the market. They also recommended that the adverse events caused by probiotics should be reported and recorded by appropriate institutions [36].

## 3. Probiotic Strains and a Literature Review of Infection Cases 

A PubMed search was performed for this review using the following keywords: “*Bifidobacterium*”, “*Lactobacillus*”, “*Bacillus*”, “*Saccharomyces cerevisiae* var. *boulardii*” or “*Saccharomyces boulardii*” and “Probiotics” and “Case Report.” Papers published in English language and reporting the cases of infections caused by probiotic strains derived from medicines, dietary supplements, or food, were selected for the review. Publications that did not clearly describe the origin of probiotic strains causing infections as well as those that did not indicate the strains found in the probiotics were excluded (Table 1). 

The evolving probiotic market and the ever-increasing demand for probiotics force us to address regulatory issues. As no strict standards are available for probiotics, they are sold as drugs, dietary supplements, or food. They are most commonly used by people who use antibiotics, those suffering from infectious diseases, or hospitalized patients [37]. At present, no generally adopted standards exist regarding the application of probiotics in patients diagnosed with organ dysfunction, immunodeficiency, and intestinal barrier dysfunction [38].

At present, Finland is the only country that conducts a detailed analysis and comparison of LAB strains obtained during bacteremia with commercially marketed probiotic strains. The National Public Health Institute (NPHI) has registered 89 cases of lactobacillemia since 1990, of which 11 were genetically indistinguishable from commercially available *L. rhamnosus* GG and most likely came from a probiotic preparation [39]. A case of bacteremia was also recorded in a patient suffering from acquired immunodeficiency syndrome (AIDS) and also Hodgkin’s lymphoma [40]. In addition, some cases of fungemia caused by the probiotic yeast *S. cerevisiae* var. *boulardii* have been reported [41].

### 3.1. Infection Cases of Probiotic Lactobacillus Strains

Bacterial species belonging to the genus *Lactobacillus* are one of the most widely used probiotics. In humans, these microorganisms are indigenous in the gastrointestinal tract and a significant part of normal vaginal microflora, mainly consisting of *Lactobacillus jensenii* and *Lactobacillus crispatus*. Due to their ability to produce lactic acid, hydrogen peroxide, and bacteriocins, it is assumed that *Lactobacillus* spp. can prevent microorganisms such as *Candida* and *Gardnerella vaginalis* from proliferating in the vagina [42,43]. The *L. rhamnosus* GG (ATCC 53103) strain is most commonly used as a probiotic and also the most frequently isolated bacteria from various infections caused by probiotic strains. It was originally isolated in 1983 by Sherwood Gorbach and Barry Goldin, and on 17 April 1985, a patent was applied by the researchers [44,45]. Gorbach and Goldin treated relapsing *C. difficile* colitis using *L. rhamnosus* GG and published the results of their study where five patients with multiple recurrences of C. difficile colitis were cured using probiotic therapy with an isolated strain [46,47,48]. In addition, *L. rhamnosus* GG was reported to reduce the duration and alleviate the symptoms of rotavirus diarrhea and may help to prevent atopic diseases among infants [47].

The literature indicates two cases of liver abscess [49,50] in the incidence of empyema in a patient who underwent lung transplantation [51], and a case of late-onset sepsis [52] and pneumonia [53]. However, endocarditis and sepsis followed by bacteremia are the most prevalent lactobacilli infections. Infectious endocarditis caused by *L. rhamnosus* GG, which was confirmed to be associated with the strain found in probiotic, was reported only in adults, of which the youngest were two persons aged 28 [54,55] and the oldest was an 80-year-old woman [56]. It was also found to be more common in men than in women. Among the affected adults, two patients aged 28 [54] and 50 [57] years did not present any comorbidities that constituted risk factors for infection, while the remaining five had bicuspid aortic valve [55], alcoholic liver cirrhosis [58], uncontrolled diabetes mellitus [59], hereditary hemorrhagic telangiectasia [48], paroxysmal atrial fibrillation, and mitral regurgitation of unknown severity [56]. Three patients who had endocarditis recovered after undergoing treatment with ampicillin and gentamicin [54,55,57], one with meropenem [59], one with penicillin and gentamicin [44], and one with amoxicillin/clavulanate and gentamicin [60]. Unfortunately, Naqvi et al. reported in 2018 that a 36-year-old woman with alcoholic liver cirrhosis died due to endocarditis caused by *L. rhamnosus* GG, although she was treated with penicillin and gentamicin [58].

Endocarditis associated with *Lactobacillus* strains was first reported in 1938 by Marchall F [61]. Infections caused by lactobacilli are difficult to treat. Studies have reported that the rate of lactobacilli endocarditis-related mortality was as high as 30% even with appropriate antibiotic therapy. Treatment with high doses of antibiotics such as penicillin or clindamycin, and in most cases glycopeptides and cephalosporins, is generally recommended for *Lactobacillus* infections [62]. The risk factors for endocarditis caused by *Lactobacillus* strains are immunocompromisation, structural cardiac disease, recently performed surgery, continuous use of antibiotics, and acute comorbidities [63].

Sepsis caused by *L. rhamnosus* GG is mainly reported in newborns [64,65,66]. Land et al. described two cases of sepsis in children: one boy (aged 6 weeks) and one girl (aged 6 years) [67]. Kochan et al. presented the case of sepsis in a woman (aged 24 years) who had aortic valve replacement [38]. The authors indicated that the patient recovered, but did not provide information about the antibiotic administered. In the case of children, the following risk factors for sepsis were found: pharmacological closure of the patent ductus arteriosus and respiratory distress syndrome [64,65], short bowel syndrome as a result of inherited intestinal atresia with volvulus [66], trisomy 18 and triple-X syndromes [65], operation repair of a double-outlet right ventricle with pulmonary stenosis, spastic paralysis, microcephalus, mental abnormality, and seizure [67]. Among the affected children, two recovered after treatment with clindamycin [65], and three children with ampicillin administered alone [67] or in combination with piperacillin and tazobactam [64] or with ceftriaxone [54]. A newborn (born in the 36th week of pregnancy) was cured by treatment with penicillin and gentamicin [67]. The increased incidence of sepsis in neonates, compared to other pediatric cases or adult cases, suggests that immune deficiency may put this population at a high risk of probiotic sepsis.

Cases with *Lactobacillus*-related bacteremia were primarily characterized by an underlying disease or immunosuppression. The risk factors for this condition include impairment in host defense mechanisms and presence of severe medical conditions, history of surgery, and long-term use of antibiotics that are ineffective against lactobacilli. So far, four cases of bacteremia caused by *L. rhamnosus* derived from the probiotic have been described in the literature. The occurrence of bacteremia has been observed in newborns [66], infants [68], and adults [25,50]. Risk factors identified for bacteremia in children include gastroschisis [54] and short gut syndrome [68], and adults include ulcerative colitis [25], diabetes mellitus, hypertension, and end-stage renal disease [50]. All patients affected with bacteremia recovered after undergoing treatment with various antibiotics. Of these, two received ampicillin in combination with ceftriaxone [66] or gentamicin [68], one received amoxicillin and clavulanate [25], and one received imipenem and vancomycin [50].

Kothari et al. indicated that sepsis is presumably related to the movement of bacteria through an impaired intestinal wall. Normally, in a healthy immunocompetent host, the translocated bacteria are caught and lysed within mesenteric lymph nodes. However, this defense response is compromised in individuals with, for example, heart failure, which compromises their gut barrier function. According to a study, intestinal wall dysfunction can be a result of splanchnic ischemia, and several human infections caused by probiotic strains are associated with mucosal transmission [69].

*Lactobacillus paracasei* and *Lactobacillus acidophilus* are widely used in probiotics for the treatment of vaginal candidiasis and diarrhea [70,71]. Reports indicate the occurrence of rare endocarditis events due to infections with these species—two by *L. paracasei* derived from a probiotic [28,72] and one by *L. acidophilus*—also from a probiotic [73]. Otto et al. described the case of *L. paracasei*-related endocarditis in a 77-year-old man who had multiple serious comorbidities such as prostate cancer, hiatal hernia, mitral insufficiency due to valvular prolapsus, hypertension, and bipolar disorder [72]. Similarly, Kato et al. reported the incidence of endocarditis due to *L. paracasei* infection in a male patient (aged 78 years) who had acute bicuspid aortic stenosis with diffuse fibrosis of the middle layer of the left ventricle [28]. The patients were cured by treatment with amoxicillin in combination with gentamicin [72] and with clindamycin, respectively [28]. In 2017, Martin et al. reported the occurrence of *L. acidophilus*-related endocarditis in a male patient (aged 57 years). The patient did not have any risk factors for infection [73]. 

One case report of intra-abdominal abscess [74] and cholecystitis [75] caused by *L. casei* and *L. fermentum*, respectively, can be found in the literature. Vanichanan et al. observed the infection in a man aged 60 years [74], and Chery et al. in a man aged 81 years [75]. In the 60-year-old patient, who had a renal transplant, *L. casei* infection was treated with vancomycin and meropenem [74]. In the 81-year-old patient who had diabetes mellitus, coronary artery disease, hypertension, and dyslipidemia, the infection caused by *L. fermentum* was cured with linezolid [75].

### 3.2. Infection Cases of Probiotic Bifidobacterium Strains

*Bifidobacterium* was the first isolated probiotic bacterium. In 1905, Henry Tissier, a French pediatrician, observed a low number of *Bifidobacterium* cells in the stool of infants with diarrhea, as compared to healthy infants, and isolated the bacterium. He suggested that *Bifidobacterium* could treat diarrhea and promote the restoration of healthy gut microflora in affected patients [4]. Bacteremia caused by probiotic *Bifidobacterium* strains seems to be uncommon and not well studied, since these microorganisms are normally regarded as contaminants and not as primary invaders [57]. The occurrence of bacteremia related to *Bifidobacterium longum* infection was shown in two premature neonates with umbilical vein varix and necrotizing enterocolitis [76], and with respiratory distress syndrome [77]. One case of sepsis related to *Bifidobacterium breve* was reported in a boy (aged 2 years) who had Philadelphia chromosome-positive B-cell acute lymphoblastic leukemia [78]. One of the affected neonates was cured by treatment with cefotaxime, vancomycin, and metronidazole followed by penicillin G [76], and the boy was treated with piperacillin/tazobactam. The other neonate also recovered, but no information was found regarding the antibiotic treatment administered [77].

### 3.3. Infection Cases of Probiotic Bacillus Strains

Probiotic substances with spores have been commercialized for applications in humans and are widely used in countries such as the USA, Australia, South America, Asia, and Europe [67,79]. In Italy, such probiotics have been used for a long time, and an Italian product (Enterogermina) containing the spores of *Bacillus clausii* in a suspension form has been sold since 1958. This product contains the spores of *B. clausii* strains OC, NR, SIN, and T, which are antibiotic-resistant [80]. However, data regarding the quality of preparations containing *Bacillus* spores are limited [81]. Literature indicates that so far two species of *Bacillus* have been isolated from cases of bacteremia—*B. clausii* [79,82] and *B. subtilis* [83]. Two reports of *B. clausii* bacteremia were identified: a 4-month-old boy who had congenital heart disease [82] and a middle-aged man with type II diabetes [79]. The patients were cured by treatment with vancomycin and teicoplanin, respectively. Oggioni et al. described the case of recurrent bacteremia due to *B. subtilis* infection in a male chronic lymphocytic leukemia patient (aged 73 years) following recovery with the use of ceftazidime, amikacin, and vancomycin [83].

### 3.4. Infection Cases of Probiotic S. cerevisiae var. boulardii

*Saccharomycescerevisiae var. boulardii* was discovered in 1920 by Henri Boulard, a French microbiologist, in IndoChina at the time of cholera epidemic. He noticed that individuals who chewed lychee or mangosteen skin or consumed a specific tea preparation were free from the disease. Later, Boulard patented the strain he isolated, and in 1947, it was traded to Biocodex company, which was formed for manufacturing the strain. The species was approved in 1953 and is the sole probiotic yeast used till now [52]. Using genotyping techniques, it has been shown that probiotic yeasts *S. boulardii* do not form a separate species, but are a subspecies (*varietas*) of *S. cerevisiae* [84]. Recently, *S. boulardii* was included as a component in probiotic supplement products used for diarrhea, even in children [85].

The use of *S. boulardii* is associated with invasive fungemia, which is also caused by another subtype of *S. cerevisiae*, especially in preterm infants, immunocompromised individuals, or patients with debilitating diseases. Of all fungemia cases, *S. cerevisiae* was responsible for 0.1–3.6% [86]. Fungemia related to *S. cerevisiae* var. *boulardii* was reported in infants [86], toddlers [87], adults [88], and those over 70 years of age [89,90], with men being more commonly affected than women. These patients generally presented with various risk factors, such as repeated antibiotic therapy [86]; hemodialysis [88]; intubation [88]; enteric nutrition [90]; and atrial fibrillation, hypertension, and coronary artery disease [89]. An almost 4-month-old boy was treated with amphotericin B, but there is no information about treatment results [86]. The other patients were cured by administering fluconazole [88] and voriconazole followed by caspofungin [88], micafungin [90], and a combination of micafungin and fluconazole [89]. Gkentzi et al. reported the incidence of fungemia without any predisposing factors in a boy (aged 2 years) who was cured by micafungin [87].

**Table 1:** Summary of the reviewed cases of infections associated with probiotics use.

Microbial Species	Infection	Age/Sex	Probiotic Species	Species Detected	Risk Factors	Treatment	Outcome	Ref.
*Lactobacillus* sp.								
*L. rhamnosus*	Endocarditis	28/M	*Lactobacillus* spp.	*L. rhamnosus*	None	Ampicillin and gentamicin	Recovered	[54]
		28/M	*Lactobacillus* spp.	*L. rhamnosus*	Bicuspid aortic valve	Ampicillin and gentamicin	Recovered	[55]
		36/F	*L. rhamnosus*, *L. acidophilus*, *S. cerevisiae*	*L. rhamnosus*	Alcoholic liver cirrhosis	Penicillin and gentamicin	Died	[58]
		40/M	*Lactobacillus* spp.	*L. rhamnosus*	Uncontrolled diabetes mellitus	Meropenem	Recovered	[61]
		50/M	*L. rhamnosus*	*L. rhamnosus*	None	Ampicillin and gentamicin	Recovered	[57]
		>65/-	*L. rhamnosus*	*L. rhamnosus*	Hereditary hemorrhagic telangiectasia	Amoxicillin/clavulanate and gentamicin	Recovered	[60]
		80/F	*L. rhamnosus*, *L. paracasei*	*L. rhamnosus*	Paroxysmal atrial fibrillation and mitral regurgitation of unknown severity	Penicillin and gentamicin	Recovered	[56]
	Sepsis	Newborn, 23rd week of pregnancy/F	*L. rhamnosus* GG	*L. rhamnosus* GG	Pharmacological closure of the patent ductus arteriosus and respiratory distress syndrome	Clindamycin	Recovered	[65]
		Newborn, 26th week of pregnancy/F	*L. rhamnosus* GG	*L. rhamnosus* GG	Respiratory distress syndrome	Ampicillin and piperacillin with tazobactam	Recovered	[64]
		Newborn, born in the 36th week of pregnancy/M	*L. rhamnosus* GG	*Lactobacillus* spp.	Short bowel syndrome due to inherited intestinal atresia with volvulus	Ceftriaxone and ampicillin	Recovered	[66]
		Newborn, born in the 39th week of pregnancy/M	*L. rhamnosus* GG	*L. rhamnosus*	Trisomy 18 and triple-X syndromes	Clindamycin	Recovered	[65]
		6 weeks/M	*L. rhamnosus* GG	*L. rhamnosus* GG	Operation repair of a double-outlet right ventricle with pulmonary stenosis	Penicillin G and gentamicin	Recovered	[67]
		6/F	*L. rhamnosus* GG	*Lactobacillus* spp.	Spastic paralysis, microcephalus, mental abnormality, and seizure	Ampicillin	Recovered	[67]
		24/F	*L. rhamnosus*	*L. rhamnosus*	Aortic heart valve replacement	No information	Recovered	[38]
	Bacteremia	Newborn, born in the 34th week of pregnancy/M	*L. rhamnosus* GG	*L. rhamnosus* GG	Gastroschisis	Ceftriaxone and ampicillin	Recovered	[66]
		15 month old/F	*L. rhamnosus* GG	*L. rhamnosus* GG	Short gut syndrome	Ampicillin and gentamicin	Recovered	[68]
		64/F	*L. rhamnosus* GG	*Lactobacillus* spp.	Ulcerative colitis	Amoxicillin/clavulanate	Recovered	[25]
		82/F	*Lactobacillus* spp.	*Lactobacillus* spp.	Diabetes mellitus, hypertension, and end stage renal disease	Imipenem and vancomycin	Recovered	[50]
	Empyema in a patient who underwent lung transplantation	56/M	*L. rhamnosus* GG	*L. rhamnosus* GG	Idiopathic pulmonary fibrosis following double-lung transplantation, gastroesophageal reflux disease, and HIV infection	No information	Recovered	[51]
	Late-onset sepsis	Newborn, born in the 25th week of pregnancy/M	*L. rhamnosus* *B. bifidum*	*L. rhamnosus*	Laparotomy	Benzylpenicillin	Recovered	[52]
	Liver abscess	74/F	*L. rhamnosus* GG	*L. rhamnosus*	Hypertension and noninsulin-dependent diabetes mellitus	Ciprofloxacin and clindamycin	Recovered	[49]
		82/F	*Lactobacillus* spp.	*Lactobacillus* spp.	Diabetes mellitus, hypertension and end stage renal disease	Imipenem and vancomycin	Recovered	[50]
	Pneumonia	11 month old/F	*L. rhamnosus* GG	*L. rhamnosus*	Trisomy 21 and a history of esophageal atresia but who was otherwise thought to be immunocompetent	Ampicillin/sulbactam	Recovered	[53]
*L. paracasei*	Endocarditis	77/M	*L. paracasei*	*L. paracasei*	Prostate cancer, hiatal hernia, mitral insufficiency due to valvular prolapsus, hypertension, and bipolar disorder	Amoxicillin and gentamicin	Recovered	[72]
		78/M	No information, daily use of probiotics	*L. paracasei*	Heart diseases	Clindamycin	Recovered	[28]
*L. acidophilus*	Endocarditis	57/M	*Lactobacillus* spp.	*L. acidophilus*	None	No information	Recovered	[73]
*L. casei*	Intra-abdominal abscess	60/M	*L. casei*, *L. plantarum*	*L. casei*	Renal transplant	Vancomycin and meropenem	Recovered	[74]
*L. fermentum*	Cholecystitis	81/M	*L. acidophilus*	*L. fermentum*	Diabetes mellitus, coronary artery disease, hypertension and dyslipidemia	Linezolid	Recovered	[75]
*Bifidobacterium* sp.								
*B. longum*	Bacteremia	Newborn 17 weeks, birthweight 1240/-	*B. longum*, *B. bifidum*, *B. breve*, *B. infantis*, *L. rhamnosus*	*B. longum*	Prematurity, umbilical vein varix, necrotizing enterocolitis	Cefotaxime, vancomycin, metronidazole then penicillin g	Recovered	[76]
		Newborn, <28 weeks birthweight <1.000 g/-	*B. longum subspecies infantis*, *L. acidophilus*	*B. longum*	Respiratory distress syndrome	No information	Recovered	[77]
*B. breve*	Sepsis	2 year old/M	*Bifidobacterium* sp., *Lactobacillus* sp.	*B. breve*	Philadelphia chromosome-positive B-cell acute lymphoblastic leukemia	Piperacillin/tazobactam	Recovered	[78]
*Bacillus* sp.								
*B. clausii*	Bacteremia	4 month old/M	*B. clausii*	*B. clausii*	Congenital heart disease	Vancomycin	Recovered	[82]
		Middle-aged/M	*B. clausii*	*B. clausii*	Type II diabetic	Teicoplanin	Recovered	[79]
*B. subtilis*	Recurrent bacteremia	73/M	*B. subtilis*	*B. subtilis*	Chronic lymphocytic leukemia	Ceftazidime, amikacin and vancomycin	Recovered	[83]
*Saccharomyces* sp.								
*Saccharomyces cerevisiae var. boulardii*	Fungaemia	3.5 month old/M	*Saccharomyces cerevisiae var. boulardii*	*Saccharomyces cerevisiae*	Repeated antibiotic therapy	Amphotericin b	No information	[86]
		2/M	*Saccharomyces cerevisiae var. boulardii*	*Saccharomyces cerevisiae*	None	Micafungin	Recovered	[87]
		25/F	*Saccharomyces cerevisiae var. boulardii*	*Saccharomyces cerevisiae*	Hemodialysis	Fluconazole	Recovered	[88]
		37/M	*Saccharomyces cerevisiae var. boulardii*	*Saccharomyces cerevisiae*	Intubation	Voriconazole and next caspofungin	Recovered	[88]
		73/F	*Saccharomyces cerevisiae var. boulardii*	*Saccharomyces cerevisiae*	Nutrition enterally	Micafungin	Recovered	[90]
		74/M	*Saccharomyces cerevisiae var. boulardii*	*Saccharomyces cerevisiae*	Atrial fibrillation, hypertension and coronary artery disease	Micafungin and fluconazole	Recovered	[89]

## 4. Conclusions

In summary, although the benefits of using probiotics are indisputable and widely proven, very rarely the side effects of their use may appear. According to the literature, some probiotic strains can cause serious adverse events such as sepsis, endocarditis, and abscesses. These exceptionally rare infections are mainly observed in the higher risk groups including patients who are immunocompromised (after organ transplants, receiving non-immunosuppressive therapies, having reduced immunity, diagnosed with AIDS, leukemia, or other neoplasms, and receiving chemotherapy); patients who are seriously and chronically ill, including those in need of central intravenous catheters and parenteral nutrition, and patients with malnutrition due to various causes; premature babies, newborns, and infants during the development of the immune system; elderly people; and pregnant women. Paradoxically, the most severely ill patients are the ones who mainly benefit from the use of probiotics, but this population is also at the highest risk of developing adverse effects. Therefore, physicians selecting probiotics for treatment should carefully consider the risks and benefits of their use and closely monitor the patient’s condition after administering them.
